# Pten in mouse vagina

**DOI:** 10.18632/oncoscience.197

**Published:** 2015-08-20

**Authors:** Shinichi Miyagawa, Taisen Iguchi

**Affiliations:** Okazaki Institute for Integrative Bioscience, National Institute for Basic Biology, National Institutes of Natural Sciences, and Department of Basic Biology, SOKENDAI (The Graduate University for Advanced Studies), Okazaki, Aichi, Japan

**Keywords:** Pten, vagina

It is well known that the phosphatase and tensin homolog deleted from chromosome 10 (Pten) is a tumor suppressor and is indispensable for epithelial cell homeostasis to prevent tumor formation. Mutations in Pten are frequently found both in sporadic and hereditary cancers, and mice with Pten deletion and/or loss-of-function mutations are highly susceptible to tumor induction by aberrant phosphatidylinositol 3-kinase (PI3K)/Akt pathway activation [1, 2].

The female reproductive tract (FRT) consists of the sites for reproduction and is of significant concern for women's health and disease, notably tumorigenesis. Despite the importance of this organ system, the molecular and cellular mechanisms that regulate cell proliferation and differentiation of these organs are not yet fully understood. Obviously, estrogen plays a central role in female reproductive organ biology. In the FRT, estrogen stimulation increases the organ weight, and promotes cell proliferation and differentiation, whereas estrogen withdrawal induces rapid involution, resulting in atrophy. These specific and reversible effects of estrogen are important in maintaining homeostasis and are required for normal health and reproduction. It is currently thought that excess estrogen stimulation plays a role in the etiology of hyperplasia and/or tumors in female reproductive organs. Therefore, homeostasis of FRT should be separately regulated according to the presence or absence of estrogen in order to prevent ectopic and excess cell proliferation, respectively.

The mouse vagina exhibits a unique feature in response to estrogens and provides an intriguing model for analyzing stratified and squamous differentiation in the epithelium, because it undergoes characteristic changes from non-keratinized to a fully keratinized epithelium depending on the levels of endogenous estrogen during the estrous cycle. In addition, the mouse model allows a manipulation of estrogen levels by ovariectomy (OVX) and exogenous estrogen treatment. The vaginae of OVX mice show atrophied epithelia of 2-3 cell layers, whereas estrogen administration rapidly induces epithelial cell proliferation in the basal layer. The suprabasal cells are no longer mitogenic but differentiate while moving up through the epithelium. Finally, apical cells exhibit keratinization. The fully stratified and keratinized vaginal epithelium resembles the typical stratified and keratinized epidermis found in the skin and other organs. Pten has a pivotal role in maintaining stratified squamous epithelia, because conditional knockout (CKO) of Pten in the epithelium leads to hyperplasia, hyperkeratosis and tumor formation in squamous tissues (e.g., squamous cell carcinomas; SCC) [3]. In our current study [4], we utilized vaginal epithelium-specific conditional Pten knockout mice to elucidate the role of Pten in the vagina.

PI3K/Akt signaling is a downstream and essential mediator for estrogen-induced events in FRT [5, 6]. We thus hypothesized that Akt activation by Pten mutation results in the phenocopy of estrogen-stimulated status and/or squamous epithelial hyperplasia even in the absence of estrogen. Pten CKO OVX mice, however, did not show such a simple phenotype, but exhibited epithelial hyperplasia in the suprabasal cells with increased cell mass and mucin production. No stratified epithelial cell markers, such as CK1 and filaggrin, were expressed. By contrast, estrogen administration to Pten CKO OVX mice induced stratification and keratinized differentiation in the vaginal epithelium as in the control mice. One of the most intriguing findings is that Pten is exclusively expressed in the suprabasal cells in the absence of estrogens, whereas estrogen administration induced Pten expression in the basal cells. Pten thus has dual roles in the vaginal epithelium, depending on the presence or absence of estrogen. In the absence of estrogen, Pten inhibits ectopic cell proliferation and abnormal mucin production in the suprabasal cells. In the presence of estrogen, Pten functions in the basal cells to prevent excessive basal cell proliferation as in the case of other squamous tissues.

The current result that Pten regulates homeostasis of the vagina in different ways is quite unique and provides new insights into how Pten functions in tissue homeostasis. Primary vaginal cancer is rare, accounting for only 1-3% of gynecological malignancies, but SCC is the most common invasive malignancy of the cervix, vagina and vulva [7]. Our findings shed light on the underlying mechanisms of cancer biology associated with the Pten/PI3K/Akt signaling pathway and its crosstalk to estrogen.
